# Evaluation of the federal hospital atlas in the context of the hospital reform 2024: a critical analysis of the healthcare provision in German hospitals

**DOI:** 10.1186/s13690-025-01639-8

**Published:** 2025-06-16

**Authors:** Jonas Roos, Theresa Isabelle Wilhelm, Ron Martin, Kristian Welle, Elio Assaf, Robert Kaczmarczyk

**Affiliations:** 1 Department of Orthopedics and Trauma Surgery, University Hospital of Bonn, Bonn, Germany; 2 Eye Center, Medical Center, Faculty of Medicine, University of Freiburg, Freiburg im Breisgau, Germany; 3 Departement of Plastic and Hand Surgery, Burn Care Center, BG Klinik Bergmannstrost Halle, Halle (Saale), Germany; 4 Department of Dermatology and Allergy, School of Medicine, Technical University of Munich, Munich, Germany

**Keywords:** Hospital reform Germany, Emergency care, Population density, Regional health disparities, German healthcare system

## Abstract

**Background:**

In the context of the ongoing German hospital reform, the newly introduced Federal Hospital Atlas aims to increase transparency regarding hospital structures and service quality. However, concerns have been raised regarding its completeness and reliability. Understanding regional differences in hospital characteristics—such as ownership type, staffing levels, emergency care provision, and specialization—is essential to assess the current state of healthcare and inform policy decisions.

**Methods:**

We conducted a cross-sectional analysis of 1,628 hospitals using data from the Federal Hospital Atlas (as of April 2025). Hospitals were categorized by ownership type, region (old vs. new federal states), population density, and specialization. Multivariable regression models—including ordinal logistic and negative binomial regressions—were used to examine associations with nursing staff ratios, emergency care participation, the number of specialized departments, and certifications.

**Results:**

Among the 1,628 hospitals analyzed, 36.57% were public, 35.27% nonprofit, and 28.16% private. Nonprofit (OR = 0.58, 95% CI [0.46–0.74]) and public hospitals (OR = 0.69, 95% CI [0.55–0.86]) had significantly lower nursing staff ratio categories compared to private hospitals but were more likely to provide emergency care (nonprofit: OR = 3.05, 95% CI [2.27–4.12]; public: OR = 4.81, 95% CI [3.52–6.61]). Trauma and/or orthopedic specialization was associated with higher emergency care participation (trauma: OR = 21.8; both: OR = 38.7) and higher structural differentiation (both: IRR = 3.11 for departments; IRR = 4.94 for certifications). Hospitals in densely populated areas had better nursing staff ratios (OR = 1.38, 95% CI [1.15–1.67]) and more certifications (IRR = 2.86, 95% CI [2.46–3.33]). No significant differences were found between old and new federal states in staffing or emergency care provision.

**Conclusion:**

The analysis reveals significant differences in hospital facilities and service provision in Germany. Public and non-profit hospitals are more heavily involved in emergency care, but have comparatively lower nursing staff ratio categories. Hospitals specializing in trauma or orthopedic care, as well as those in urban areas, tend to be larger and better equipped. These results point to imbalances and underscore the need for political action to ensure equal access and high-quality hospital care across regions and providers.

**Table:** 

**Text box 1. Contributions to the literature**
• There is limited research on the structural implications of the 2024 German hospital reform and the role of the new Federal Hospital Atlas.
• This study reveals disparities in emergency care provision and nurse staffing across hospital ownership types and population densities.
• These findings highlight the importance of improving health data governance and communication for informed policy-making and patient empowerment.

## Introduction

The hospital reform initiated by Health Minister Karl Lauterbach represents a pivotal shift in the German healthcare system. The Hospital Care Improvement Act (Krankenhausversorgungsverbesserungsgesetz, KHVVG) is based on the recommendations of the “Commission for a Modern and Demand-Oriented Hospital Care System”, which were published on December 6, 2022 [1]. This hospital reform is designed to achieve three primary objectives: securing and enhancing treatment quality, ensuring comprehensive medical coverage, and reducing administrative burden.

 [2]. A major structural shift is being considered, which would entail the termination of the existing case-based reimbursement model and the introduction of standby payments as a novel financial foundation for hospitals in Germany [3]. These payments will be distributed to hospitals for the provision of essential services, with the aim of covering approximately 60% of hospital operating costs in the future [4]. Additionally, improvements are planned for the existing emergency care system. To facilitate more expedient patient access to appointments, service centers will be expanded and integrated with emergency dispatch centers. Medical associations will be required to ensure round-the-clock telemedicine services and home visits, while Integrated Emergency Centers (Integrierte Notfallzentren - INZ) with fixed opening hours and digital networking will be established throughout the country. Both the INZ and mobile emergency services will also be authorized to issue medical leave certificates [5]. In essence, the introduction of standby payments is intended to alleviate the economic pressures on hospitals by securing funding for essential hospitals regardless of specific service delivery.

In 2008, the “Weisse Liste” (White List), an online portal for patients offering information on hospitals, physicians, and nursing homes, was introduced [6]. The website was managed by the umbrella associations of patient and consumer organizations in collaboration with the Bertelsmann Foundation [7]. In March 2024, the “Weisse Liste” was discontinued as part of the hospital reform and replaced by the “Klinik-Atlas” [8]. The new atlas has its origins in the Hospital Transparency Act, which requires the publication of data pertaining to the structure and performance of hospitals [9]. On May 17, 2024, the Federal Ministry of Health (BMG) launched the interactive Federal Hospital Atlas [10]. However, the atlas has already drawn substantial criticism from the medical community, as it lacks layperson-friendly language and relies heavily on the international ICD-10/11 and OPS codes [11]. Consequently, on May 30, 2024, the AWMF Ad-hoc Commission on Health Care Structures urged the Federal Ministry of Health to label the website as a test version due to incomplete and preliminary nature of data, to prevent patient misdirection [12].

Even today, there are still health differences between the federal states as well as in the salary structure [13, 14]. For this reason, this study has paid particular attention to the differences in health care structures based on population density and between the old and new federal states.

This study aims to critically analyze hospital structures and care provision in Germany using data from the newly introduced Federal Hospital Atlas, with a focus on regional and ownership-related disparities in emergency care, staffing, and specialization. The findings are discussed in light of the 2024 hospital reform objectives to contribute to current health policy debates. The study will analyze the distribution of ownership, nursing staff ratios, specialized departments, and certifications, as well as their impact on care quality, with a particular focus on structural and organizational disparities between hospitals in the old states of Germany compared to the new states. A particular focus is on orthopedics and trauma surgery as a relevant specialist department in emergency care and in hospitals. The findings aim to contribute to improving the transparency and effectiveness of healthcare provision in Germany.

## Methods

### Data source

The data for this study are drawn from the Federal Hospital Atlas, published by the Federal Ministry of Health (BMG) on May 17, 2024. The atlas includes comprehensive information on 1,628 hospitals across Germany, including treatment cases, nursing staff ratios, the number of nursing personnel involved in direct patient care, hospital ownership, postal codes, certifications, specialty departments, and emergency care levels. The certifications, selected by the Institute for Quality Assurance and Transparency in Healthcare (IQTIG), are intended to provide high-quality indicators of treatment quality [15]. As the IQTIG methodology is still under development, the certifications represent a preliminary selection. The dataset was downloaded on April 14, 2025.

### Data processing and cleaning

To analyze regional differences, the data were categorized into the “new” federal states (Brandenburg, Mecklenburg-Vorpommern, Saxony, Saxony-Anhalt, Thuringia) and the “old” federal states (all remaining federal states) based on postal codes. A simplified definition of population density from the Federal Statistical Office was employed [16]; however, the criterion “directly adjacent to a densely populated area” was omitted due to technical limitations. Within the same locality, the average population density was calculated for all postcode areas, ensuring that sparsely populated postcode areas within densely populated cities were still classified as “densely populated.”

For the population and area data used to calculate population density, we consulted [17]. This source uses the latest population data from federal and state agencies and raw data from OpenStreetMap [18], last updated on July 15, 2023.

### Statistical analyses

All statistical analyses were conducted using the R programming language, version 4.4.1 [19]. Specific analyses relied on the MASS package (version 7.3–61) for negative binomial models [20], the ordinal package (version 2023.12-4) for cumulative link models [21], and the gtsummary package (version 1.7.2) for presenting results [22].

For descriptive analysis, hospital characteristics were summarized using counts (n) and percentages (%) for categorical variables. Continuous variables were described using medians and interquartile ranges (IQR: Q1– Q3), chosen due to potential non-normal distributions.

To investigate the associations between hospital characteristics (independent variables) and the selected outcomes (dependent variables), we employed multivariable regression models tailored to the nature of each dependent variable. Four main dependent variables were examined in our models. First, the Nursing Staff Ratio was treated as an ordinal variable, categorized into five quantiles (quintiles) ranging from “far below average” to “far above average.” This categorization was informed by practical guidelines and the potential for non-linear relationships in staffing metrics.

 [15, 23], aiming to provide interpretations relevant to policy and management benchmarks while acknowledging the inherent simplification. Second, emergency care participation was operationalized as a binary variable. Third and fourth, the number of specialized departments and the number of selected certifications were both treated as count variables. The independent variables included in the models were hospital ownership type (using private hospitals as the reference category), region (comparing new to old federal states as the reference), population density (comparing medium and densely populated areas to sparsely populated areas as the reference), and the level of trauma/orthopedic specialization (comparing hospitals with orthopedic only, trauma only, or both specializations to those with none as the reference). These predictors were selected based on their established relevance in health services research concerning hospital structure, resources, and service provision within the German context, as well as data availability.

For the ordinal outcomes (nursing staff ratio category and emergency care participation), cumulative link models (CLMs) with a logit link function were fitted. These models estimate the cumulative odds of a hospital falling into a higher category of the outcome versus a lower one, conditional on the predictor variables. A key assumption underlying the CLM is the proportional odds assumption, which posits that the association between predictors and the odds of crossing any specific category threshold remains constant across all thresholds. The results from these models are presented as Odds Ratios (ORs). An OR exceeding 1 suggests that an increase (or presence) of the predictor is associated with higher odds of being classified in a higher outcome category, relative to the reference group, whereas an OR less than 1 indicates lower odds. For the count outcomes (number of departments and number of certifications), negative binomial regression models were employed. This modeling approach was chosen over standard Poisson regression to appropriately handle potential overdispersion, where the variance in the count data exceeds the mean, a common characteristic in healthcare data. These models estimate the expected count of the outcome conditional on the predictors, utilizing a logarithmic link function. The results are reported as Incidence Rate Ratios (IRRs). An IRR greater than 1 signifies that the expected count of the outcome is higher by a factor equivalent to the IRR for a given predictor group compared to its reference group. Conversely, an IRR less than 1 indicates a lower expected count.

Missing data were addressed using listwise deletion. This means that for any given regression model, observations containing missing values on any variable included in that specific model were excluded from that analysis. This approach relies on the assumption that data are missing completely at random (MCAR), which may be a strong assumption in the context of healthcare data collection; potential biases introduced by this method represent a limitation of the study. The final number of observations included in each model is explicitly reported with the regression results in Tables 2 and 3.

For all models, we report the estimated ORs or IRRs along with their corresponding 95% confidence intervals (CIs), which provide a measure of the precision of the estimate. P-values are also reported to assess the statistical significance of the observed associations, with a conventional threshold of *p* < 0.05 used to denote statistical significance.

### AI-assisted writing

During the writing process, AI-supported tools (GPT-4o, Claude 3 Sonnet) were utilized to ensure efficient and precise articulation. The authors meticulously reviewed the results to verify correctness and accuracy.

## Results

### Distribution of ownership

This study examined emergency care provision across different types of hospital ownership in Germany. Of the 1,628 hospitals analyzed, 1616 hospitals have had ownership information. 36.57% (*n* = 591) were public, 35.27% (*n* = 570) were non-profit, and 28.16% (*n* = 455) were privately owned. The median number of nurses per hospital was 131 (IQR 58–247). The number of cases treated per year varied widely among hospitals, with a median of 7,304 cases per year (IQR 2,633 − 14,072).

### Emergency care levels

From 1,575 hospitals, that had emergency care information provided, 71.6% (*n* = 1127) were providing emergency care. The majority of hospitals were located in sparsely populated areas (defined as areas with < 50,000 inhabitants and/or < 100 inhabitants/km²). Approximately a quarter (*n* = 391, 24%) of hospitals were located in the new federal states.

### Regional differences in ownership and staffing

In the old federal states, there was a higher proportion of non-profit hospitals (36%) and a slightly lower proportion of public hospitals (37%) compared to the new federal states (32% non-profit and 36% publicly operated). The median number of nurses was similar across regions (old states: 131, new states: 130). The median annual treatment cases were also comparable (old states: 7,355; new states: 7,172).

### Emergency care participation and specialty departments

Hospitals in the new federal states were more likely to be located in sparsely populated areas (67% in new states vs. 58% in old states; see Table 1 for details). A higher proportion of hospitals that had neither trauma nor orthopedic specializations was found in the west federal states (57%) compared to the new federal states (53%).

**Table 1:** General overview for *n* = 1628 hospitals from the federal hospital atlas and breakdown by old and new federal States (as of April 14, 2025)

Characteristic	*N*	Overall *N* = 1,628^*1*^	Old Federal States *N* = 1,237^*1*^	New Federal States *N* = 391^*1*^
**Hospital Ownership**	1,616			
public		591 (37%)	451 (37%)	140 (36%)
non-profit		570 (35%)	446 (36%)	124 (32%)
private		455 (28%)	331 (27%)	124 (32%)
Unknown		12	9	3
**Nursing Staff**	1,628	131 (58–247)	131 (57–253)	130 (64–235)
**Nursing Staff Ratio Category**	1,610			
far below average		322 (20%)	243 (20%)	79 (20%)
below average		288 (18%)	227 (19%)	61 (16%)
average		311 (19%)	239 (20%)	72 (19%)
above average		367 (23%)	268 (22%)	99 (26%)
far above average		322 (20%)	246 (20%)	76 (20%)
Unknown		18	14	4
**Annual Treatment Cases**	1,628	7,304 (2,624–14,072)	7,355 (2,649–14,230)	7,172 (2,503–13,278)
**Treatment Cases Category**	1,628			
very few		323 (20%)	246 (20%)	77 (20%)
few		325 (20%)	246 (20%)	79 (20%)
medium		327 (20%)	247 (20%)	80 (20%)
many		326 (20%)	248 (20%)	78 (20%)
very many		327 (20%)	250 (20%)	77 (20%)
**Provides Emergency Care**	1,628			
Yes		1,127 (69%)	860 (70%)	267 (68%)
No		448 (28%)	340 (27%)	108 (28%)
Unknown		53 (3.3%)	37 (3.0%)	16 (4.1%)
**Population Density Category**	1,628			
sparsely populated		984 (60%)	721 (58%)	263 (67%)
medium population density		23 (1.4%)	20 (1.6%)	3 (0.8%)
densely populated		621 (38%)	496 (40%)	125 (32%)
**Trauma/Orthopedic Specialization**	1,628			
none		935 (57%)	726 (59%)	209 (53%)
ortho		297 (18%)	204 (16%)	93 (24%)
trauma		294 (18%)	238 (19%)	56 (14%)
both		102 (6.3%)	69 (5.6%)	33 (8.4%)
**Departments**	1,628	5 (2–9)	5 (2–9)	5 (2–9)
**Certificates**	1,628	1 (0–3)	1 (0–3)	0 (0–2)

^*1*^n (%); Median (Q1– Q3)

### Factors influencing nursing staff ratio and emergency care provision

To investigate the characteristics associated with hospital nursing staff ratio categories and emergency care participation levels, we employed ordinal logistic regression (cumulative link models). The results, including odds ratios (ORs), 95% confidence intervals (CIs), and p-values, are presented in Table 2. An OR greater than 1 indicates higher odds of a hospital being classified in a higher category of the respective ordinal outcome (Nursing Staff Ratio or Emergency Care Participation), while an OR less than 1 indicates lower odds, relative to the reference group. Compared to private hospitals (reference group), non-profit hospitals were associated with lower odds of being in a higher Nursing Staff Ratio Category (OR = 0.58, 95% CI [0.46–0.74], *p* < 0.001) but higher odds of participating in higher levels of emergency care (OR = 3.05, 95% CI [2.27–4.12], *p* < 0.001). Similarly, public hospitals were associated with lower odds of being in a higher Nursing Staff Ratio Category (OR = 0.69, 95% CI [0.55–0.86], *p* = 0.001) and significantly higher odds of participating in higher levels of emergency care (OR = 4.81, 95% CI [3.52–6.61], *p* < 0.001) relative to private hospitals.

Regarding specialization, relative to hospitals with no trauma or orthopedic specialization (reference group), those with only orthopedic specialization showed no statistically significant association with Nursing Staff Ratio Category (OR = 1.12, 95% CI [0.88–1.41], *p* = 0.4) but were linked to higher odds of participation in higher emergency care levels (OR = 3.48, 95% CI [2.50–4.90], *p* < 0.001). Hospitals with trauma specialization showed an association approaching statistical significance with lower odds for Nursing Staff Ratio Category (OR = 0.80, 95% CI [0.63–1.01], *p* = 0.056) and substantially higher odds for higher emergency care participation levels (OR = 21.8, 95% CI [11.6–46.6], *p* < 0.001). Hospitals specializing in both trauma and orthopedics also showed a tendency towards lower odds for Nursing Staff Ratio Category (OR = 0.73, 95% CI [0.51–1.05], *p* = 0.088) alongside markedly higher odds for higher emergency care participation levels (OR = 38.7, 95% CI [12.0–238], *p* < 0.001).

No statistically significant associations were observed between geographic region (New vs. Old Federal States [reference group]) and either Nursing Staff Ratio Category (OR = 1.03, 95% CI [0.84–1.26], *p* = 0.8) or Emergency Care Participation level (OR = 0.94, 95% CI [0.70–1.26], *p* = 0.7).

Considering population density, compared to hospitals in sparsely populated areas (reference group), those in medium population density areas showed no significant association with either outcome (Nursing Staff Ratio Category: OR = 1.06, *p* = 0.9; Emergency Care Participation: OR = 2.76, *p* = 0.2). However, hospitals located in densely populated areas were associated with higher odds of being in a higher Nursing Staff Ratio Category (OR = 1.38, 95% CI [1.15–1.67], *p* < 0.001), but showed no statistically significant association with Emergency Care Participation level (OR = 0.81, 95% CI [0.62–1.07], *p* = 0.14).

**Table 2:** Factors influencing nursing staff ratio and emergency care provision

	Nursing Staff Ratio Category				Emergency Care Participation			
**Characteristic** ^*1*^	**N** ^*1*^	**OR** ^*1*^	**95% CI** ^*1*^	**p-value** ^*1*^	**N** ^*1*^	**OR** ^*1*^	**95% CI** ^*1*^	**p-value** ^*1*^
**Ownership**	1,598				1,575			
private		—	—			—	—	
non-profit		0.58	0.46–0.74	**< 0.001**		3.05	2.27–4.12	**< 0.001**
public		0.69	0.55–0.86	**0.001**		4.81	3.52–6.61	**< 0.001**
**Trauma/Orthopedic Specialization**	1,598				1,575			
none		—	—			—	—	
ortho		1.12	0.88–1.41	0.4		3.48	2.50–4.90	**< 0.001**
trauma		0.80	0.63–1.01	0.056		21.8	11.6–46.6	**< 0.001**
both		0.73	0.51–1.05	0.088		38.7	12.0–238	**< 0.001**
**Region**	1,598				1,575			
Old Federal States		—	—			—	—	
New Federal States		1.03	0.84–1.26	0.8		0.94	0.70–1.26	0.7
**Population Density**	1,598				1,575			
sparsely populated		—	—			—	—	
medium population density		1.06	0.50–2.25	0.9		2.76	0.67–18.9	0.2
densely populated		1.38	1.15–1.67	**< 0.001**		0.81	0.62–1.07	0.14

^*1*^For the ordinal dependent variables nursing staff ratio category and emergency care participation, a cumulative link model was employed. Odds ratios (ORs) are provided such that an OR > 1 indicates higher odds of being in a higher category of the outcome, while an OR < 1 indicates lower odds. The estimation strategy relies on the assumption of proportional odds and involves categorizing continuous variables based on theoretical considerations and data distribution; a more detailed explanation of these decisions, as well as the identification strategy and underlying assumptions, is available in the Methods section. Missing data were excluded from the analysis (listwise deletion), resulting in a reduction in the sample size, as indicated by the observation counts in the table

Abbreviations: CI = Confidence Interval, OR = Odds Ratio

We investigated the associations between hospital characteristics and the number of specialized departments and selected certifications using negative binomial regression models, where an IRR greater than 1 signifies that the expected count of departments or certifications is higher for that group compared to the reference group, while an IRR less than 1 signifies a lower expected count. Compared to private hospitals (reference group), non-profit hospitals were associated with a higher rate of specialized departments (IRR = 1.14, 95% CI [1.05–1.25], *p* = 0.003) and a higher rate of selected certifications (IRR = 1.79, 95% CI [1.46–2.19], *p* < 0.001). Public hospitals showed associations with even higher rates relative to private hospitals for both departments (IRR = 1.60, 95% CI [1.48–1.75], *p* < 0.001) and certifications (IRR = 2.96, 95% CI [2.44–3.60], *p* < 0.001). Hospitals with trauma and/or orthopedic specialization demonstrated associations with higher rates of both outcomes compared to those with no such specialization (reference group). Specifically, orthopedic specialization was associated with higher rates (Depts: IRR = 1.80, 95% CI [1.65–1.97], *p* < 0.001; Certs: IRR = 2.49, 95% CI [2.05–3.03], *p* < 0.001). Trauma specialization showed associations with higher rates (Depts: IRR = 2.52, 95% CI [2.32–2.74], *p* < 0.001; Certs: IRR = 3.94, 95% CI [3.27–4.75], *p* < 0.001), and specialization in both trauma and orthopedics was associated with the highest rates observed (Depts: IRR = 3.11, 95% CI [2.75–3.52], *p* < 0.001; Certs: IRR = 4.94, 95% CI [3.76–6.57], *p* < 0.001).

Regarding geographic location, hospitals in the new federal states were associated with a slightly higher rate of specialized departments compared to those in old federal states (reference group) (IRR = 1.09, 95% CI [1.01–1.18], *p* = 0.028). However, no statistically significant association was found between region and the number of selected certifications (IRR = 1.05, 95% CI [0.89–1.26], *p* = 0.554). Considering population density relative to sparsely populated areas (reference group), hospitals in medium population density areas showed an association bordering on statistical significance for a higher rate of departments (IRR = 1.31, 95% CI [1.00–1.71], *p* = 0.050) and a statistically significant association with a higher rate of certifications (IRR = 2.13, 95% CI [1.23–3.92], *p* = 0.011). Hospitals situated in densely populated areas were associated with significantly higher rates of both specialized departments (IRR = 1.46, 95% CI [1.36–1.56], *p* < 0.001) and selected certifications (IRR = 2.86, 95% CI [2.46–3.33], *p* < 0.001, Table 3).

**Table 3:** Factors influencing the number of specialized departments and selected certifications

	Number of Departments				Number of Certificates			
**Characteristic** ^*1*^	**N** ^*1*^	**IRR** ^*1*^	**95% CI** ^*1*^	**p-value** ^*1*^	**N** ^*1*^	**IRR** ^*1*^	**95% CI** ^*1*^	**p-value** ^*1*^
**Ownership**	1,616				1,616			
private		—	—			—	—	
non-profit		1.14	1.05–1.25	**0.003**		1.79	1.46–2.19	**< 0.001**
public		1.60	1.48–1.75	**< 0.001**		2.96	2.44–3.60	**< 0.001**
**Trauma/Orthopedic Specialization**	1,616				1,616			
none		—	—			—	—	
ortho		1.80	1.65–1.97	**< 0.001**		2.49	2.05–3.03	**< 0.001**
trauma		2.52	2.32–2.74	**< 0.001**		3.94	3.27–4.75	**< 0.001**
both		3.11	2.75–3.52	**< 0.001**		4.94	3.76–6.57	**< 0.001**
**Region**	1,616				1,616			
old federal states		—	—			—	—	
new federal states		1.09	1.01–1.18	**0.028**		1.05	0.89–1.26	0.554
**Population Density**	1,616				1,616			
sparsely populated		—	—			—	—	
medium population density		1.31	1.00–1.71	**0.050**		2.13	1.23–3.92	**0.011**
densely populated		1.46	1.36–1.56	**< 0.001**		2.86	2.46–3.33	**< 0.001**

^*1*^Negative Binomial Regression models were employed for the count-based dependent variables (Number of Departments, Number of Certificates). Incidence Rate Ratios (IRRs) are provided; an IRR > 1 indicates that the expected count of the outcome (departments or certifications) is higher for that group by a factor equal to the IRR, relative to the reference group. Conversely, an IRR < 1 indicates a lower expected count relative to the reference group. The estimation strategy relies on assumptions appropriate for count data models (e.g., regarding the mean-variance relationship and independence, detailed in Methods). Further information on the identification strategy, the rationale for the selection and categorization of variables, and a full description of the underlying assumptions is available in the Methods section. Missing data were excluded from the analysis using listwise deletion, resulting in the sample size (N) indicated for each model

Abbreviations: CI = Confidence Interval, IRR = Incidence Rate Ratio

This table presents the results of Negative Binomial Regressions analyzing factors influencing the number of specialized departments and selected certifications in German hospitals. Predictors include hospital ownership type, regional location (old vs. new federal states), trauma/orthopedic specialization (none, ortho, trauma, or both) and population density (sparse, medium, or dense). Incidence Rate Ratios (IRR) are reported with corresponding 95% confidence intervals (CI) and p-values.

## Discussion

This study provides a comprehensive overview of the current healthcare landscape in Germany based on data from the Federal Hospital Atlas. Among the 1,628 included hospitals, there is an almost equal distribution nonprofit (36%) and public hospitals (36.57%), while private hospitals represent a slightly lower proportion (28%). Compared to the German Federal Statistical Office’s total of 1,893 hospitals, this represents a discrepancy of 265 hospitals (16.3%) not captured in the Hospital Atlas [24]. Furthermore, compared to the 2022 hospital statistics, there is a notable discrepancy, particularly in the number of private facilities (756 vs. 455) [25]. Even considering the approximately 66 hospital closures from 2020 to 2023, the discrepancy remains remarkable [26], indicating that a considerable percentage of hospitals may not be represented in the Federal Hospital Atlas.

Nonprofit and public hospitals had a worse nursing staff ratio categories compared to private hospitals in our model, which may reflect financial constraints or indicate a divergence from the standard staffing model. This is particularly notable given that public hospitals typically have higher personnel expenses compared to private hospitals [27] and, according to our models, also provide coverage for a broader range of specialized departments and certifications. These discrepancies underscore the importance of further investigation into structural and organizational factors to develop strategies for improving care quality. Private hospitals, in comparison, may appear to achieve higher efficiency in material and personnel resource allocation [28]. Additionally, our findings show that the nursing staff ratio categories is significantly worse in sparsely populated regions compared to densely populated areas, pointing to more severe staffing shortages and distinct cost structures in rural areas. This trend may be attributable to both structural factors—such as proximity to workplaces in urban hospitals—and individual-level determinants. These associations warrant further investigation to better understand the underlying reasons.

The analysis revealed significant findings regarding disparities in emergency care levels across different hospital ownership types. Private hospitals had a lower level of emergency care provision compared to nonprofit and public hospitals in our models. This suggests that private hospitals are less involved in emergency care than nonprofit and public institutions. This discrepancy may be attributed to the financial unviability of comprehensive emergency services, particularly for smaller hospitals under private ownership. With increasing demand from patients with low-urgency cases, this can result in care deficits and economically significant costs that are often not fully compensated [29, 30]. Future standby financing could relieve hospitals of the pressures associated with mandatory service provision [31]. The variations in emergency care levels highlight the need to investigate structural and organizational differences further to ensure equitable and high-quality emergency care coverage.

Our findings indicate a clear association between orthopedic and trauma specialization and hospital participation in emergency care. Hospitals with either orthopedic or trauma specialization showed substantially higher odds of being involved in emergency care services, with trauma-specialized hospitals exhibiting particularly pronounced effects (OR = 21.8), and those with both specializations demonstrating the highest odds (OR = 38.7). This trend underscores the central role of specialized surgical services in ensuring acute care coverage, particularly in regional or supra-regional emergency networks.

Interestingly, hospitals with trauma or combined trauma/orthopedic specialties were not associated with higher nurse staffing ratio categories despite their greater involvement in emergency care. Given the nature of emergency care, where trauma-related injuries are among the most common reasons for admission [32], the higher involvement of these hospitals in emergency care is consistent with their clinical focus. However, the staffing ratios in these hospitals do not appear to match the expected intensity of care for such cases. This raises concerns about potential understaffing, particularly in facilities that regularly treat complex and highly acute emergency patients. As the staffing ratio takes into account both patient volume and care intensity, this observation could indicate that current staffing levels do not fully meet the increased demands on nursing staff in these facilities.

This structural mismatch between high emergency care responsibility and relatively low nurse staffing capacity in trauma and orthopedic hospitals has direct relevance for the 2024 hospital reform. One of the central pillars of the reform is the introduction of standby payments intended to stabilize the financial base of hospitals that provide essential services. Our results suggest that such funding mechanisms must go beyond formal service availability and incorporate indicators of care intensity and clinical specialization. Hospitals with trauma and orthopedic focus bear a disproportionate emergency burden, yet currently lack adequate staffing support. If reform measures aim to ensure sustainable quality in acute care, they must consider not just whether emergency services are provided, but how intensively they are used and resourced. This could justify differentiated staffing thresholds or earmarked funding for high-intensity specialties, particularly in rural areas where structural shortages are most pronounced.

Furthermore, trauma and orthopedic specialization was also significantly associated with a greater number of specialized departments and certifications. This reflects a higher structural differentiation and quality assurance level, possibly driven by participation in trauma networks [33] and the need for documented competence in specialized care provision, but could also reflect the fact that these hospitals are larger and more organized. The high certification levels, particularly among hospitals with combined O&U specialization (IRR for certifications = 4.94), may also contribute to enhanced visibility in health service planning and potentially greater access to funding opportunities under the reform.

## Conclusion

The objective of the hospital reform is to enhance treatment quality and ensure comprehensive medical coverage. Our analysis highlights notable discrepancies in emergency care provision and nursing staff ratios depending on hospital ownership. These findings emphasize the imperative for further research and structural adjustments to ensure the accessibility and quality of medical care across Germany. However, given the descriptive nature of this study, the results should be interpreted as an exploratory comparison rather than causal evidence. Acknowledging the study’s limitations, future research should incorporate more comprehensive analytical approaches to account for confounding factors driving differences between hospital providers.

### Limitations

This study has several limitations. First, the analysis is based on data from the Federal Hospital Atlas, which is considered a test version due to the incomplete and preliminary data. Second, not all hospitals in Germany are included, which may bias the results. Third, the nursing staff ratios reflect a single point in time and do not account for temporal changes or seasonal fluctuations. Fourth, the potential influence of factors such as regional differences in funding or specific management strategies was not subjected to a comprehensive analysis. Additionally, our study does not control for potential confounding factors, such as variations in disease burden, regional healthcare needs, or socio-economic differences, which may significantly influence hospital staffing, specialization, and emergency care participation.

## Data Availability

No datasets were generated or analysed during the current study.

## References

[1] BMG [Internet]. [cited 2024 Jun 9]. Bundeskabinett beschließt Krankenhausreform. Available from: https://www.bundesgesundheitsministerium.de/presse/pressemitteilungen/krankenhausreform-kabinett-pm-15-05-24.html

[2] BMG [Internet]. [cited 2024 Jun 7]. Krankenhausreform. Available from: https://www.bundesgesundheitsministerium.de/themen/krankenhaus/krankenhausreform

[3] BMG [Internet]. [cited 2024 Jun 9]. Krankenhausreform: Bund und Länder einigen sich auf Eckpunkte. Available from: https://www.bundesgesundheitsministerium.de/ministerium/meldungen/krankenhausreform-eckpunkte.html

[4] PK. Deutscher Bundestag. [cited 2024 Jun 19]. Große Krankenhausreform mit Vorhaltepauschalen. Available from: https://www.bundestag.de/presse/hib/kurzmeldungen-1009104

[5] BMG [Internet]. [cited 2024 Jun 9]. Lauterbach legt Eckpunkte zur Notfallreform vor. Available from: https://www.bundesgesundheitsministerium.de/presse/pressemitteilungen/eckpunkte-notfallverorgung-pm-16-01-24.html

[6] Relaunch - Weisse. Liste mit neuem Auftritt [Internet]. [cited 2024 Jun 11]. Available from: https://www.bertelsmann-stiftung.de/de/unsere-projekte/weisse-liste/projektnachrichten/weisse-liste-mit-neuem-auftritt

[7] Kuhrt N, Lindenberg F. Gesundheitsökonomen fordern Qualitätsmessung im System. Der Spiegel [Internet]. 2013 Feb 12 [cited 2024 Jun 11]; Available from: https://www.spiegel.de/wissenschaft/medizin/gesundheitsoekonomen-fordern-qualitaetsmessung-im-system-a-882665.html

[8] Ende für Weisse Liste. Start für den interaktiven Klinik-Atlas​| heise online [Internet]. [cited 2024 Jun 11]. Available from: https://www.heise.de/news/Ende-fuer-Weisse-Liste-Start-fuer-den-interaktiven-Klinik-Atlas-9687970.html

[9] BMG [Internet]. [cited 2024 Jun 11]. Krankenhaustransparenzgesetz. Available from: https://www.bundesgesundheitsministerium.de/service/gesetze-und-verordnungen/detail/krankenhaustransparenzgesetz.html

[10] BMG [Internet]. [cited 2024 Jun 11]. Bundesweiter Klinik-Atlas geht online. Available from: https://www.bundesgesundheitsministerium.de/presse/pressemitteilungen/bundesweiter-klinik-atlas-geht-online.html

[11] Bundesklinikatlas. Kritik an Nutzerfreundlichkeit [Internet]. [cited 2024 Jun 11]. Available from: https://www.aerzteblatt.de/archiv/239613?rt=c1173e330e5250a22b78bda8a335572b

[12] Ärzteblatt DÄG Redaktion Deutsches. Deutsches Ärzteblatt. 2024 [cited 2024 Jun 11]. Bundesklinikatlas: Ad-hoc-Kommission der AWMF fordert Ausweisung als Testversion. Available from: https://www.aerzteblatt.de/nachrichten/151809/Bundesklinikatlas-Ad-hoc-Kommission-der-AWMF-fordert-Ausweisung-als-Testversion

[13] Lampert T, Ziese T, Kurth BM. Gesundheitliche Entwicklungen und Trends in Ost-und Westdeutschland. 2010.

[14] Pflegeberufe [Internet]. [cited 2025 Mar 11]. Available from: https://www.lohnspiegel.de/pflegeberufe-13899.htm

[15] Methodik [Internet]. [cited 2024 Jun 19]. Available from: https://bundes-klinik-atlas.de/methodik/

[16] Statistisches Bundesamt [Internet]. [cited 2024 Jun 24]. Grad der Verstädterung. Available from: https://www.destatis.de/DE/Themen/Branchen-Unternehmen/Gastgewerbe-Tourismus/Glossar/grad-verstaedterung.html

[17] PLZ Download. • Postleitzahlen als Liste und Karte [Internet]. [cited 2025 Apr 15]. Available from: https://www.suche-postleitzahl.org/downloads

[18] OpenStreetMap [Internet]. [cited 2025 Apr 15]. OpenStreetMap. Available from: https://www.openstreetmap.org/

[19] R: The R Project for Statistical Computing [Internet]. [cited 2025 Apr 15]. Available from: https://www.r-project.org/

[20] Venables WN, Ripley BD. Modern applied statistics with S. 4th ed. New York: Springer; 2002. (Statistics and computing).

[21] Stata Programs for. Data Analysis [Internet]. [cited 2025 Apr 15]. Available from: https://stats.oarc.ucla.edu/stata/ado/analysis/

[22] Sjoberg DD, Whiting K, Curry M, Lavery JA, Larmarange J. Reproducible summary tables with the Gtsummary package. R J. 2021;13(1):570.

[23] Katalog zur Risikoadjustierung für Pflegeaufwand (Pflegelast-Katalog). Version 2024, InEK GmbH [Internet]. [cited 2024 Jun 19]. Available from: https://www.g-drg.de/archiv/pflegepersonaluntergrenzen/pflegepersonaluntergrenzen-2023/katalog-zur-risikoadjustierung-fuer-pflegeaufwand-pflegelast-katalog-version-2024

[24] Statistisches Bundesamt [Internet]. [cited 2024 Jun 19]. Krankenhäuser. Available from: https://www.destatis.de/DE/Themen/Gesellschaft-Umwelt/Gesundheit/Krankenhaeuser/_inhalt.html

[25] Statistisches Bundesamt [Internet]. [cited 2024 Jun 19]. Krankenhäuser 2023 nach Trägern und Bundesländern. Available from: https://www.destatis.de/DE/Themen/Gesellschaft-Umwelt/Gesundheit/Krankenhaeuser/Tabellen/eckzahlen-krankenhaeuser.html

[26] Bündnis Kbilanziert. Stockende Krankenhausreform, grassierender Klinikkahlschlag– Gemeingut in BürgerInnenhand [Internet]. 2023 [cited 2024 Jun 19]. Available from: https://www.gemeingut.org/buendnis-klinikrettung-bilanziert-stockende-krankenhausreform-grassierender-klinikkahlschlag/

[27] PricewaterhouseCoopers PC. [cited 2024 Jun 19]. PwC-Studie: Krankenhaus-Vergleich 2019. Available from: https://www.pwc.de/de/gesundheitswesen-und-pharma/krankenhaeuser/pwc-studie-krankenhaus-vergleich-2019.html

[28] PricewaterhouseCoopers PC. [cited 2024 Jun 19]. PwC-Studie: Krankenhaus-Vergleich 2020. Available from: https://www.pwc.de/de/gesundheitswesen-und-pharma/krankenhaeuser/pwc-studie-krankenhaus-vergleich-2020.html

[29] Walter G, Faust M, Wenske S, Raane M, Umgelter K, Schmid RM, et al. Immobilität Als Grund der vorstellung in einer krankenhausnotaufnahme?? Notfall Rettungsmed. 2024;27(4):273–80.

[30] Woschek M, Schindler CR, Sterz J, Störmann P, Willems L, Marzi I, et al. Aufnahmediagnose prellung: ätiologie, epidemiologie und Kostenfaktoren. Z Gerontol Geriatr. 2020;54(8):802. 33337522 10.1007/s00391-020-01828-w PMC8636411

[31] Ärzteblatt DÄG Redaktion Deutsches. Deutsches Ärzteblatt. 2023 [cited 2024 Jun 19]. Vorhaltefinanzierung: PKV-Gutachten warnt vor Unterversorgung und Wartelisten. Available from: https://www.aerzteblatt.de/nachrichten/145871/Vorhaltefinanzierung-PKV-Gutachten-warnt-vor-Unterversorgung-und-Wartelisten

[32] Biberthaler P, Förschner L, Gehring C, Trentzsch H, Kanz KG, Prückner S. Stellenwert der unfallchirurgie für die Notaufnahmen einer Deutschen Millionenstadt– Eine auswertung von 524.716 notfallpatienten. Unfallchirurg. 2019;122(1):44–52. 30402692 10.1007/s00113-018-0577-5

[33] AUC. TraumaNetzwerk [Internet]. [cited 2025 Apr 15]. Available from: https://www.traumanetzwerk-dgu.de/en/

